# Thriving beyond survival: Understanding utilization of perinatal health services as predictors of birth registration: A cross-sectional study

**DOI:** 10.1186/s12914-014-0038-3

**Published:** 2014-12-21

**Authors:** Michelle Jackson, Putu Duff, Santi Kusumaningrum, Lindsay Stark

**Affiliations:** 1 Program on Forced Migration and Health, Columbia University Mailman School of Public Health, 60 Haven Ave B-4 Suite 432, New York, 10032 NY USA; 2 School of Population and Public Health, University of British Columbia, 608-1081 Burrard St, Vancouver, V5N 1P5 BC Canada; 3 Center on Child Protection at the University of Indonesia, Gedung Nusantara II (Ex PAU Ekonomi) FISIP, Lantai 1, Kampus UI, Depok 16424 Indonesia

**Keywords:** Birth registration, Child protection, Health services utilization, Indonesia, Civil registration and vital statistics (CRVS)

## Abstract

**Background:**

There are an estimated 35 million unregistered children in Indonesia. To understand ways to best leverage existing health system-related resources and ensure greater protective measures for these vulnerable children, this study explores the predictive relationship between the utilization of perinatal health services and birth certificate ownership in two Indonesian provinces.

**Methods:**

This study employed a cross-sectional design with interviewer-administered household surveys to heads of households in West Nusa Tenggara and East Nusa Tenggara from May to July of 2013. The primary outcome of interest was birth certificate ownership among children under the age of 5 years old. Bivariate and multivariable regression analyses using Generalized Estimating Equations (GEE) considered a set of covariates that represented child and household socio-demographic characteristics along with health services utilization variables during pregnancy and post-pregnancy periods.

**Results:**

389 heads of households were interviewed, yielding data on a sample of 451 children under the age of 5. Fewer than 28% of children in this sample possessed a birth certificate. Nearly 57% (n = 259) of children were delivered in a clinical facility, though only 36% (n = 93) of these were legally registered. Of children born in the home (n = 194), registration dropped to 16% (n = 31). Adjusted analyses accounting for socio-demographic factors suggest that children born in a clinic facility (AOR = 2.33, 95% CI: 1.27, 4.33), hospital (AOR = 2.38, 95% CI: 1.12, 5.09), or in the presence of a skilled birth attendant (AOR = 2.35, 95% CI: 1.31, 4.23) were significantly more likely to be registered. Children whose mothers sought post-natal care were 2.99 times more likely to possess a birth certificate (AOR = 2.99, 95% CI: 1.1, 7.57). Pre-natal care was not associated with birth registration.

**Conclusion:**

These findings suggest that use of perinatal health services increases the likelihood of registering a child’s birth despite a lack of formal integration of vital registration with the health sector. Formally leveraging existing community-based health workers and perinatal services may serve to further increase registration rates in hard to reach areas of Indonesia.

## Background

There is a global crisis of invisibility impeding the protection of children worldwide [1]. An estimated 230 million – or nearly half – of the world’s children under 5 years old remain unregistered and unaccounted for through formal systems [2]. The majority of these children reside in underserved areas of Asia and Sub-Saharan Africa, and nearly one-sixth live in Indonesia alone [2],[3]. Within these countries, socioeconomic, regional and urban-versus-rural factors further affect registration rates [2]. In Indonesia, for example, fewer than 65% of children are properly registered with the state, and only 57% possess a physical birth certificate [4]. Registration rates decrease to 41% among those in the poorest quintile of the population, and to 34% for children living in rural and difficult to access areas [4]. Coverage and access also remain highly variable and disparate among Indonesia’s provinces [5]. In Yogyakarta, nearly 90% of children are registered with the government compared to fewer than 30% of children in the poorer provinces of East Nusa Tenggara, Papua, Maluku, and West Sulawesi [5],[6]. This disparity in registration rates illustrates a pattern of disadvantage and inequitable access and delivery of services seen across Indonesia and many other developing settings [7],[8].

Considerable research has shown that birth registration and possession of a birth certificate^a^ can guarantee a host of entitlements and protections that are fundamental to a child’s healthy development and wellbeing [1],[9]-[25]. Registered legal status can ensure access to state-provided benefits such as healthcare, education and social assistance, and provide long-term legal protections for formal employment and land ownership [1],[9]-[12]. In many cases, birth registration can also reduce risks to human security such as unlawful child labor practices and trafficking [2],[26]. In a recent study by the International Organization for Migration (IOM), Indonesia was identified as a key source of trafficked individuals in Southeast Asia. One in four individuals in this trafficked population were children, with the greatest risk factor for their exploitation being a lack of proper registration [26]. Accurate birth data further enables a government to equitably allocate resources and social services for the most vulnerable, and to track population health trends such as child mortality [1],[2],[9],[10],[27]. Regardless of the context, the absence of formal registration and legal identity can exacerbate existing disadvantage, and result in harmful and lasting consequences in children’s lives.

While the improvement of birth registration systems has consistently remained an important priority from a child’s rights and protection perspective, a new wave of attention around improved vital registration systems has emerged in the discourse of the Millennium Development agenda. The Millennium Development Goals (MDGs) have drawn global attention to reducing child mortality and improving maternal health care practices such as birthing with a skilled attendant present or within a health facility [27],[28]. In Indonesia, a country long criticized for inconsistencies in the implementation of maternal and child health services, the government committed itself to population-wide health strategies aimed at reaching the most vulnerable. Between 1989 and 1997, the population with access to a health professional increased from 35% to 97%, and child deaths decreased significantly [8],[9],[29]-[31].

This MDG framework has also established standardized indicators and targets for the reduction of child deaths and improved health system solutions to reach highly vulnerable populations. Accurate data to assess the progress of these important targets remains incomplete and, in some cases, is lacking entirely [12],[14],[27]. Several recent reports have strongly emphasized that without accurate records of births and population figures, particularly those in already difficult to reach areas, countries cannot properly measure their progress towards reduced maternal and child mortality with confidence [2],[12],[14],[27]. In this way, a need for better child survival data has indirectly fueled the impetus for greater-protective measures for children.

A growing body of research, primarily grey literature, attempts to understand the reasons behind low registration rates, both from supply-side deficiencies on the national-level and demand-driven barriers faced by households and individuals, in an effort to inform interventions to improve coverage. Despite increased funding and attention from international agencies, however, inhibiting factors persist, including poor political will and legislative commitments, a lack of public socialization of registration benefits and procedures, inadequate financial and human resources, and difficulty in reaching geographically isolated populations that could benefit most from formal registration [1],[2],[9],[12],[14],[16].

In Indonesia, while birth registration is a compulsory and guaranteed right per the ratification of the Child Protection Law (23/2002), the implementation of this right remains inconsistent and highly ambiguous across provinces [32]. A subsequent population law passed in 2006 by the Ministry of Home Affairs structured a passive registration system whereby parents are expected to report births at their local district or sub-district offices [32]. There is no obligation for health officials to facilitate this registration of births, though some midwives will personally facilitate the process at cost. In other cases, districts hospitals will include a birth certificate in their delivery package for an additional cost, despite that registration is free within a child’s first 60 days [32]. The registration process for children over 60 days requires that a parent or guardian pay late registration fines [32]. The Government of Indonesia has recently passed a series of legislative policies and judicial reforms that resulted in abolished late registration fines in an effort to ensure that all children under the age of 18 are registered free of cost [32]. However, it failed to officially redact the fine structure, which is still legally binding and enforced by many districts as a means of income. It also did little to reduce the cumbersome requirements required to register a birth, which include: a birth information letter from a doctor or birth attendant (Surat Keterangan Lahir); an ID of a birth witness; a family card listing both parents; an ID card for both parents; a photocopy of the parents’ marriage certificate; and a completed form requesting a birth certificate [32]. For children whose origins are unknown, a police statement is required in lieu of parental documentation [32].

The high associated costs, long distances and complicated procedures required to pursue this process continue to preclude poorer families in remote from registering the births of their children [32]. The administrative and transportation costs alone for registering a child in a largely impoverished province like East Nusa Tenggara (NTT), for example, can cost up to 6 times the average household’s monthly income [32]. Indonesia is not alone, as a recent UNICEF study shows high direct and indirect costs to be the most cited barrier by individuals in as many as 20 developing countries across the globe [2],[24].

UNICEF, as the leading technical organization supporting national action plans for countries with weak vital registration systems, has targeted strategies towards improving the political will and subsequent legislative reforms needed for nationwide change [24]. Integrating birth registration as part of the formal health systems has proven a particularly successful tactic in some target countries, particularly when coupled with outreach campaigns and the elimination of registration fees [14],[16]-[18],[24]. In Ghana, for example, the integration of birth registration into community based health mechanisms served to increase registration rates from 41% to 77% over five years, and significantly reduced the indirect costs associated with distances to registrar offices [14]. In Venezuela, the Ministry of Health incorporated registrars into clinics and hospitals specifically serving Afro-descendent communities where registration was lowest, resulting in a 17% increase in registration among this target population [24].

While research around the impact of under-registration is emerging, the majority of the empirical studies focus on the benefits of and need for adequate registration systems [1],[9],[10],[15]. There is a notable dearth of peer-reviewed evidence surrounding actual predictors associated with birth certificate ownership, particularly in Asia where vast under-registration continues to be unaddressed. The few studies that attempt to link health care utilization and birth registration have primarily done so to highlight the benefits of proper surveillance mechanisms for improved monitoring of child health outcomes or as programmatic evaluations of specific initiatives implemented by international agencies [14],[16]. One study conducted in several Latin American countries found a clear relationship between health services and birth registration, though ultimately failed to offer evidence-based recommendations for increased registration services at national and subnational levels [12]. As such, few studies have explored the clear opportunities to link these two sectors and identify which aspects of the health system might be leveraged to increase registration.

This study explores the relationship between current perinatal health services utilization and birth certificate ownership in remote and rural areas of Indonesia. Perinatal heath services refer to the care of the woman and child before, during and after delivery, and include prenatal care, care during delivery and postnatal care. Further, our study attempts to identify opportunities to leverage committed resources in the maternal and child health sectors in order to integrate permanent, protective registration measures and ensure better outcomes for vulnerable Indonesian children. In light of the existing evidence surrounding civil registration and services access, a positive association between health services utilization and birth certificate ownership was hypothesized at the outset of the study.

## Methods

### Data and measures

This analysis draws on baseline data collected across 26 villages in West Nusa Tenggara (NTB) and East Nusa Tenggara (NTT) as part of the Australian Government’s Australian-Indonesian Partners for Justice’s (AIPJ) Legal Identity Program. Indonesia’s Center on Child Protection at the University of Indonesia led the baseline study from May to July of 2013, supported by researchers from Columbia University.

#### Study setting and population

NTB and NTT provinces were targeted for this study as their populations share characteristics most representative of unregistered populations, namely high poverty, rural contexts and low human development indicators^b^. Within these provinces, eight districts were randomly assigned into a future intervention group, while seven districts were assigned to a non-intervention group and matched to intervention sites on poverty and registration rates. Using 2013 census lists, two villages from each of the 13 districts, or 26 villages in total, were randomly selected for inclusion into the study. Because the baseline assessment collected data prior to the implementation of any intervention, for the purposes of this analysis the distinction between the 16 intervention and 10 non-intervention villages is inconsequential.

#### Sample size and selection

A minimum sample size of 385 children was calculated, assuming an alpha of .05, 80% power and design effect of two, and drawing on 2011 census data indicating a 57% prevalence of birth certificate ownership among children living in NTT and NTB provinces. The total sample included 451 observations.

Eligibility criteria required households to have at least one child under the age of 5 who had slept in the home the night prior to the interview, eliminating the potential for double counting a child within a given community; and a respondent over the age of 16 years old. Female heads of households were preferred when possible, as it was presumed that these women would have more complete information about their child’s health history and their own use of perinatal health services [33],[34]. In circumstances where households had multiple children under 5, a maximum of two children were selected. Children were randomly assigned by the enumerator (without prior knowledge of the child’s birth certificate status) and selected for inclusion into the study in order to mitigate interviewer fatigue and reduce household clustering effects.

Households were randomly selected to participate in the survey using a multi-stage cluster sampling approach. Google Earth was used to defined enumeration areas and determine random start houses in each village. Subsequent households were selected by going to the next nearest household until the required number of data points had been collected. If enumerators encountered a household where no one was present, no one met inclusion criteria or if a respondent refused to participate, that household was skipped and recorded, and the next nearest household was surveyed. When possible, interviewers returned to homes where no one had been present.

#### Interviewers

Experienced enumerators were selected from local non-governmental organizations (NGOs) working in the child protection field. These individuals spoke local languages and possessed many of the socio-cultural characteristics of the target population, which helped ensure high quality data across the diverse provinces. All enumerators participated in a two-day intensive training workshop that covered unique qualities of the survey instrument, including the appropriate execution of skip patterns and the cognitive meaning of all survey questions. All enumerators were required to administer the survey with members of the research team before field-testing.

#### Instrument

The interviewer-administered household survey consisted of 111 questions drawn from a combination of internationally and nationally validated survey instruments, including Indonesia’s SUSENAS (census) and SPKBK (a longitudinal cohort study on female headed households administered by PEKKA, an NGO for the Empowerment of Female Heads of Household in Indonesia). The survey elicited the following information: socio-demographics (e.g., age, gender, education); access and utilization of health care services; access to social services; whether household members had birth and marriage certificates; barriers experienced by household members in obtaining birth and marriage certificates; child health outcomes (e.g., child mortality); and knowledge and perceptions surrounding legal identity documents.

In each province, the survey was field tested in two villages outside of the study’s sampling frame to assess the cognitive understanding and interpretation of specific questions. Based on field-testing, the survey was modified to improve the reliability and consistency of respondent answers. Each survey took between 30 to 60 minutes to complete.

#### Measures

For this analysis, the primary outcome of interest is birth certificate ownership, measured by whether or not a child had a birth certificate (self-report by the head of household). The primary independent variables included the following perinatal health care services: utilization of prenatal and postnatal care; place of delivery, defined as hospital, clinic/health post or home; presence of a Skilled Birth Attendant (SBA) at delivery. The use of prenatal and postnatal care was dichotomized based on a “yes” or “no” response to the questions: “Did you access [prenatal/postnatal] care by a health care provider [before/after] the delivery of this child?” Association between method of payment for health services, defined as state-subsidized *Jamkesmas*, out-of-pocket expenditure or other (capturing private insurance) and birth certificate ownership were also examined. Method of payment served as a related income measure to identify the poorest households due to eligibility criteria of government-subsidized health insurance [35]. Covariates and potential confounders include: the head of household’s age, sex, marital status, educational attainment (defined as having attended no school, primary school, high school, or high school or higher); and child’s age and sex. Household religion was also collected, though it is common knowledge that NTB residents are predominantly Muslim while those in NTT are largely Catholic. The ages of adult heads of household and children were measured continuously. These demographic variables were considered potential confounders as they have previously been shown to be associated with health outcomes and birth registration [2],[36].

#### Ethical considerations

The Legal Identity Program was granted an Institutional Review Board exemption through the Child Protection in Crisis Network at Columbia University and the Center for Child Protection (PUSKAPA) at the University of Indonesia. Subjects were informed of the study purpose and asked to give explicit consent prior to their participation.

### Analysis

Data were analyzed using SPSS Version 21 and SAS 9.4. All bivariate and multivariable analyses were performed using Generalizing Estimating Equations (GEE) with a logit link for the dichotomous outcome to account for the cluster effect of multiple children in a household. Standard errors for estimates were adjusted using a working correlation structure. Unadjusted Odds ratios (OR), Adjusted Odds Ratios (AOR) and 95% Confidence Intervals (95% CIs) for the dichotomous variables were calculated using GEE bivariate and multivariable logistic regressions.

Multivariable logistic regression models were subsequently constructed to determine the relationship between perinatal health utilization variables and birth certificate ownership. Statistically significant (at p < 0.05) variables in bivariate analysis that were also identified as *a priori* as confounders in the literature were considered for inclusion in the multivariable models. Literacy and ever school were excluded from the multivariable model due to collinearity with education. A backward stepwise approach was used to construct the models. The Quasi-likelihood Information Criteria (QIC) value determined model fit with the final multivariable model exhibiting the lowest QIC value.

## Results

### Sample characteristics

The 451 children included in this analysis, all of whom were under 5 years old, were evenly distributed by sex and province, and averaged 2 years of age (median = 2.0, IQR = 1.0-3.0) (see Table 1 for sample characteristics). Fifty one percent (n = 230) were Muslim, 35% Catholic (n = 159), 12% (n = 55) Protestant and 2.2% (n = 10) reported a range of different religions. As expected, province and religion were inextricably linked as nearly 100% of Muslims lived in NTB and 100% Christians in NTT. Consequently, religion was not considered in the adjusted analysis, as province accounted for religion and other contextual and procedural factors distinctive of each province.

**Table 1:** **Household- and child-level characteristics and unadjusted associations with birth certificate ownership among 451 children in East and West Nusa Tenggara (NTT and NTB)**

Characteristic	Total n = 451 (100%)	Yes BC n = 124 (27.5%)	No BC n = 327 (72.5%)	Odds ratio (95% CI)
**Respondent’s sex** ^**†**^				
Male	117 (25.7%)	32 (27.6%)	**84 (72.4%)**	1.04 (0.64 – 1.69)
Female	335 (74.3%)	92 (27.5%)	**243 (72.5%)**	REF
Respondent’s age, median years (IQR)	31.0 (27–37)	32.0 (28–38)	31.0 (27–37)	1.00 (0.98-1.02)
Child’s age, mean years (IQR)**	2.0 (1–3)	2.37 (1–3)	1.83 (0.8-3)	1.06 (1.03 - 1.10)
Respondent married^†^*	410 (90.9%)	119 (29.2%)	288 (70.8%)	3.52 (1.20 - 10.27)
**Child’s sex** ^**‡**^				
Male**	235 (47.9%)	63 (27.0%)	170 (73.0%)	0.91 (0.60 – 1.38)
Female**	216 (52.1%)	59 (27.7%)	154 (72.3%)	REF
**Respondent education level** ^**†**^				
High school or higher**	111 (24.6%)	54 (48.6%)	57 (51.4%)	14.03 (4.08 - 48.18)
Middle School**	100 (22.0%)	28 (28.0%)	72 (72.0%)	5.83 (1.66 - 20.49)
Primary School*	193 (42.5%)	39 (20.5%)	151 (79.5%)	3.87 (1.37 - 13.18)
No School	48 (10.6%)	3 (6.3%)	45 (93.8%)	REF
Respondent ever attended school^†^	421 (93.3%)	122 (29.0%)	299 (71.0%)	5.53 (1.29 - 23.78)
Respondent literate^†^**	399 (88.7%)	120 (30.1%)	279 (69.9%)	6.69 (2.03 - 22.04)
**Province** ^**‡**^				
West Nusa Tenggara	227 (50.3%)	91 (40.1%)	136 (59.9%)	REF
East Nusa Tenggara**	224 (49.7%)	33 (14.7%)	191 (85.3%)	3.88 (2.42 - 6.23)
**Religion** ^**‡**^				
Muslim**	227 (50.3%)	90 (39.6%)	137 (60.4%)	REF
Catholic**	159 (35.3%)	28 (17.6%)	131 (82.4%)	0.33 (0.20 - 0.54)
Protestant	55 (12.2%)	5 (9.1%)	50 (90.9%)	0.15 (0.06 - 0.39)
Other	10 (2.2%)	1 (10.0%)	9 (90.0%)	0.19 (0.02 - 1.15)
Payment for health services^‡^				
Other**	23 (5.1%)	13 (56.6%)	10 (43.5%)	7.82 (2.44 - 20.05)
Out of pocket**	256 (57.0%)	83 (32.4%)	173 (67.6%)	2.95 (1.76 - 4.95)
JAMKESMAS	166 (36.6%)	24 (14.5%)	142 (85.5%)	REF

*Statistically significant at p < 0.05.

**Statistically significant at p < 0.01.

^†^While 389 Households are included in this sample, these figures refer to an individual child’s caregiver or head-of-household and may be counted multiple times adjusted through Generalized Estimating Equations (GEE).

^‡^Child-level demographics out of the total sample of 451 children <5 years.

### Birth certificate ownership

Birth certificate ownership in NTB and NTT was low with only 27.5% of children reported to own one. This decreased to 20%^c^ when accounting for only those children that could show proof of ownership to interviewers. Additionally, only 14% of children under one year old had a birth certificate. This analysis also revealed regional inequities in birth certificate ownership. Over forty percent (40.1%) of children in NTB province had a birth certificate compared to only 14.7% among children living in the neighboring province of NTT.

Univariate analyses revealed a host of factors to be associated with birth certificate ownership. As presented in Table 1, the social and demographic characteristics, education and literacy of the caregiver, religion, province, marital status and a child’s age were significantly associated with birth certificate ownership.

### Utilization of maternal and child health care services

Data on four types of utilization of health services around child birth – prenatal care, postnatal care, facility-based delivery and skilled attendant at birth – indicate that an overwhelming majority of women interact with the health care system^d^ before, during and after the birth of their children (Table 2). Ninety-four percent (n = 480) of children in our sample benefited from accessing at least one form of perinatal health service in the course of their mother’s pregnancy, and 50% benefited from at least three of the four measured types of utilization. Nearly 95% (n = 415) of births benefited (at least once) from prenatal services and 83% (n = 363) from at least one post-natal care visit; both services are typically sought through either formally trained or traditional midwives.

**Table 2:** **Percentage of population seeking perinatal health services or engaging in health services utilization**

	N = 451	%
**Place of delivery**		
*Hospital*	*72*	*15.9%*
*Clinic*	*187*	*41.2%*
*Home*	*195*	*43.0%*
Skilled attendant present	259	57.0%
Sought prenatal care	415	94.3%
Sought postnatal care	363	82.9%

### Maternal and child health-related predictors of birth certificate ownership

After adjusting for the household’s method of payment for health services and province of residence, the respondent’s level of education, and child’s age, findings suggest that interactions with the formal health system and the utilization of health services continue to significantly influence birth registration status (Table 3). In multivariable analysis, children delivered in a clinic setting (AOR = 2.33, 95% CI: 1.27, 4.33) or hospital (AOR = 2.38, 95% CI: 1.12, 5.09) were more likely to be registered than those delivered in the home. Similarly, children born in the presence of a skilled birth attendant were 2.35 times (95% CI: 1.31, 4.23) more likely to have their births registered. For children whose mothers sought post-natal care, there was a 2.99 greater likelihood of owning a birth certificate (95% CI: 1.19, 7.57).

**Table 3:** **Unadjusted and adjusted associations between utilization of perinatal health care services and birth certificate ownership for children <5, using Generalized Estimating Equations and Logistic Regression (n = 451)**

	Unadjusted odds ratios			Adjusted odds ratios		
	OR	95% CI	*p* -value	OR	95% CI	*p* -value
Place of delivery^*†*^						
Hospital	2.64	(1.39-5.02)	0.003*	2.38	(1.12-5.09)	0.025*
Clinic	2.93	(1.77-4.83)	<0.001*	2.33	(1.27-4.33)	0.007**
Home	REF	-	-	REF	-	-
Skilled attendant at birth^‡^^*†*^	2.85	(1.76-4.59)	<0.001*	2.35	(1.31-4.23)	0.004*
Sought prenatal care^*†*^	1.89	(0.65-5.52)	0.246	-	-	-
Sought postnatal care^*†*^	3.73	(1.73-8.06)	0.001*	2.99	(1.19-7.57)	0.020*

^†^*Statistically significant at p < 0.05.

^†^Odds ratio represents association with birth certificate ownership; Adjusted for Education of caregiver, Province, Payment Method for Health and Child’s Age.

^‡^Skilled Birth Attendant (SBA) is defined as a Physician, Trained Midwife or Nurse. This demonstrates the odds of receiving a birth certificate if an SBA is present at the birth. This is compared to being attended to by a Traditional Birth Attendant (dukun) or being alone.

NB: Each row represents a separate multivariable model, adjusted for Education of caregiver, Province, Payment Method for Health and Child’s Age.

## Discussion

An average of only 27.5% of children from the sample in West Nusa Tenggara (NTB) and East Nusa Tenggara (NTT) provinces possessed a birth certificate, despite a nationally established civil registration system with provincial mandates for universal registration. Provincially, over forty percent (40.1%) of children in NTB province had a birth certificate compared to only 14.7% among children living in the neighboring province of NTT. These figures are well below Indonesia’s national average of 57%, reflecting the existing under-registration among Indonesia’s rural and impoverished regions [4]. This failure to ensure greater registration coverage reflects a need for more diverse and cross-sectoral approaches to Indonesia’s poorest and most remote areas. Nearly 95% of the respondents in the sample utilized some form of health service during pregnancy or as part of their post-delivery routine. Those children delivered in a clinic setting, in the presence of a birth attendant or whose mothers sought post-natal care were all significantly more likely to possess a birth certificate. These findings highlight some important ways forward towards strengthening registration processes.

Over the past few decades, the Government of Indonesia has committed to developing population-wide health strategies aimed at reaching its most vulnerable populations. In 1989, when maternal and child mortality rates were 440 and 72 per 100,000 live births respectively, Indonesia implemented a village-based midwife program targeted specifically at women in hard to reach rural villages [29],[30]. This program trained 54,000 midwives over 7 years, which successfully increased the number of women giving birth with a skilled birth attendant, and established high use of antenatal and perinatal care. Indonesia also established 8,000 community health centers (*puskesmas*) in rural areas to provide primary health care and a wide spectrum of maternal care services, and to ensure the implementation of the Integrated Management of Childhood Illness (IMCI) strategy [7]. By 1997, 97% of Indonesia’s population had access to these midwives and to basic maternal and child health care [7],[8],[29],[30]. These initiatives created the physical infrastructure and human resource capacity necessary to ensure greater vital registration [24]. Despite the resources that have been invested in maternal health and child survival programming and Indonesia’s commitment to improved vital registration, however, there have been limited attempts to utilize this existing infrastructure to overcome low registration rates in the protection sector.

### Considerations for increased registration

Indonesia’s registration process faces challenges similar to those that historically plagued the country’s maternal and child health sector: high costs, limited access, low knowledge and inconsistent and decentralized implementation [32]. Further findings from this study presented elsewhere found that direct and indirect costs were the largest cited barriers in registering births [32]. For low-income or resource-constrained populations, the costs associated with transportation, food and loss of livelihood made the process prohibitively expensive. A burdensome process that required excessive documentation including a formal notification of birth, a family card, parents’ marriage certificate, and two witnesses was found to create additional barriers. Additionally, among non-Muslim populations (i.e. NTT province), marriage certificates can only be acquired at the Dinas Kependudukan dan Catatan Sipil office (SDUKCAPIL) at the district level (as opposed to the sub-district Kantor Urusan Agama [KUA], or Office of Religious Affairs for Muslims), further increasing the administrative and indirect costs required to obtain proper documentation in advance of a child’s registration [32]. As a result, these findings suggest that bringing *integrated* and *permanent* registration services directly to rural communities is more likely to be successful. Incorporating active registration in existing health services may increase access for households that are burdened by the current process [24].

#### Integrate formal registration within health clinics

Findings show that in even the most impoverished and least developed provinces of Indonesia, the vast majority of women access some form of maternal or child health service at least once before, during and after the birth of their children. This health care utilization manifests as interactions in clinical settings with trained midwives and through home visits with traditional birth attendants or healers. Services range from pre-natal care to immunization and follow-up visits for the children themselves. Those women who delivered in a clinic setting or in the presence of a skilled birth attendant, regardless of their social or demographic characteristics, were significantly more likely to register their children, despite the lack of formal integration of these two sectors. This suggests that despite a woman’s educational attainment and other contextual factors, clinic settings and skilled health workers already play a significant role in increasing registration among underserved populations of women. This is likely due to information shared by individual health providers and the direct provision of the SKL card (birth notification card), the first requisite document required to complete the birth registration process. Those women that gave birth by traditional birth attendant or in their own homes are required to travel to a clinic for a notification of birth, thus creating one more barrier in the registration process.

Findings also show that not all women who use health services, even formal services, are registering their children. Results found that of the 57% (n = 257) of children delivered in a clinical facility, 36% (n = 93) were registered. This suggests possible missed opportunities to eliminate procedural and cost barriers, which may currently prohibit greater uptake. This also implies that women who do not interact with the formal health care system may be precluded from the benefits facilitated through clinics or through trained birth attendants, such as the issuance of the birth notification card (SKL). Nearly one third of women in our sample delivered their children in their own homes, and 43% relied on the use of a traditional birth attendant during delivery. These women, whom a passive registration system will likely miss, may be systematically excluded from registering their children simply through circumstance.

Through both community-based facilities and outreach health workers, however, health services are almost universally available, and there are opportunities to build on this infrastructure to increase birth registration. In Ghana, a country with similar compulsory laws and barriers to registration as Indonesia, a birth registration overhaul led to significant increases in registration rates between 2004 and 2008 [14],[33]. With political and legislative will and limited funding, the government implemented a comprehensive program that included free registration periods, volunteer-led birth registration days, mobile registration efforts and community health worker integration [24],[34]. Registration offices were moved into community-based health facilities and linked to district wide records, directly connecting health care and registration at birth [14].

In Indonesia, the Pusat Kajian Perlindungan Anak (PUSKAPA) at the University of Indonesia in partnership with the Austrialian-Indonesian Partnership for Justice (AIPJ) are piloting an Integrated Services initiative to assist poor families in the free facilitation of the birth registration process and its requisite documents. This program, though it relies on sporadic single-day campaigns that assist a maximum of 50 couples per village, creates a mobile village-level civil registrar system for free marriage legalization (through a Court representative), registration of marriage and issuance of a marriage certificate (with a Religious Affair officer for Muslim couples and a Civil Registrar for non-Muslim couples), and the registration of birth and issuance of a birth certificate (also through the Civil Registrar officer). It also aims to provide fee and fine waivers for the poor and marginalized to remove yet another structural barrier affecting a poor and disadvantaged population.

Building on these “integrated” efforts and a surge of permanent community health clinics (*puskesmas*) specifically implemented to access difficult to reach populations, an adapted model could be envisioned for hard to reach areas of Indonesia. Such an approach would leverage the Integrated Services model and incorporate birth registration within these *puskemas* to provide permanent and accessible services closer to the populations they intend to reach. The integration of vital events registration with the health system could also incentivize greater utilization of these centers, particular promoting greater use of post-natal care and immunization services.

#### Leverage existing human resources in health

Introducing a cadre of 54,000 skilled village midwives to rural and remote villages represents one of the most important strategies that the Government of Indonesia has instituted in an effort to increase access to health and improve dismal infant and maternal mortality rates [29]-[31]. These skilled health care workers provide maternal and primary health care services to those previously impeded from such services due to distance and cost, and often provide services directly in a patient’s home.

Community-based curriculums led by local health care providers could also be expanded to support registration practices. For example, as part of efforts to encourage better parenting practices, a ‘Mother Class’ initiative has been developed to help meet the Millennium Development Goals for maternal and child mortality. In these classes, midwives demonstrate and teach parenting techniques to new mothers, and emphasize the importance of immunizations, breastfeeding and early childhood development. As part of the curriculum, increasing awareness around the importance of birth registration is a key component [27]. These Mother Classes are not systematically implemented, however; and even in areas where they do exist, many times the structural factors discussed above prevent households from registering their children.

A comprehensive and integrated delivery model should embody a systems approach that encourages clear policies, standards and trainings while also relying on community-based participation and demand, making greater coverage possible [7]. This system would attempt to increase awareness about birth registration before and after delivery, integrate preventative child protection measure within in the first 30 days of a child’s life and leverage existing health services utilization and human resources.

### Limitations

Given the eventual evaluative nature of the larger study, provinces, districts and villages were purposefully selected and matched, thus limiting the overall generalizability of the results. While NTT and NTB may be characteristic of many of Indonesia’s poor and largely rural provinces, care should be taken when interpreting results for other populations. In addition, the cross-sectional nature of the study design limits findings to correlations and associations precluding temporal inference, though the sequencing of events (e.g., a child’s birth precedes their registration) serves to somewhat mitigate this bias. The household survey also relied on self-report for many measures, potentially introducing recall or reporting biases. In particular, the registration of a birth could be seen as a socially desirable behavior, and may lead to an overestimate of the prevalence of registered children. To mitigate this risk, certain validation techniques, including a request to demonstrate physical birth certificate documents, were used to confirm self-report data. Additionally, interviews were conducted in private rooms and respondents were assured that all information shared would remain confidential in an effort to encourage honest responses. Given that our independent variable was binary and based on ‘no’ versus any use of prenatal and postnatal care, the frequency of pre- and post- natal care and its effects on birth certificate ownership was not assessed. Future research that examines the frequency of health services utilization may be helpful in identifying a dose–response relationship between the frequency of perinatal health services access and birth certificate ownership.

Lastly, because the outcome was relatively common (28%), logistic regression likely overestimated the effect of these predictors on the outcome of interest. Relative risk regression on future longitudinal data may help to reduce this effect.

## Conclusion

Birth registration is an important and undervalued process that can help countries monitor progress towards improved health outcomes, and help the most underserved break harmful cycles of disadvantage. Despite the benefits, the majority of children in East and West Nusa Tenggara remain unregistered. The Millennium Development agenda has considerably increased global attention to combatting child mortality and resulted in greater demand for better data and indicators [27],[28]. The improved health systems and human resource capacity that have been developed in response, however, have thus far failed to extend their efforts to ensure greater preventative child protection measures through the systematic registration of births. Our findings suggest an opportunity to promote a greater systems approach to child protection in Indonesia through the integration of birth registration into health services. Integrating services into community clinic settings and with community health workers and birth attendants already serving the community may help overcome existing barriers and improve registration rates among some of the most vulnerable populations in Indonesia.

### Endnotes

^a^UNICEF defines birth registration and birth certificates distinctly, where *birth registration* is the continuous, permanent and universal recording within the civil registry of the occurrence and characteristics of births in accordance with the legal requirements of a country. *Birth Certificate* is a vital record that documents the birth of a child.

^b^NTT Province (HDI = 68.28,% rural = 80.7%, 20.24% under province-specific poverty line of 234,141rp/month) and NTB Province (HDI = 66.89,% rural = 68.3%, 17.25% of population under the province-specific poverty line of 263,107rp/month) compared to Indonesia’s averages (HDI = 73.29,% rural = 50%, 11.47% living under the country poverty line of 275,779rp/month) [37].

^c^This figure specifically accounts for birth certificate ownership and not for registration.

^d^The health care system comprises formally recognized health providers (trained midwives, physicians, nurses, etc.) as well as traditional birth attendants and *dukuns*.
